# Traditional and novel time-series approaches reveal submarine groundwater discharge dynamics under baseline and extreme event conditions

**DOI:** 10.1038/s41598-021-01920-0

**Published:** 2021-11-19

**Authors:** Tristan McKenzie, Henrietta Dulai, Peter Fuleky

**Affiliations:** 1 Department of Earth Sciences, University of Hawaiʻi at Mānoa, Honolulu, HI 96822 USA; 2 Department of Economics, University of Hawaiʻi at Mānoa, Honolulu, HI 96822 USA; 3 Present Address: Department of Marine Sciences, University of Gothenburg, Gothenburg, Sweden

**Keywords:** Environmental sciences, Hydrology, Ocean sciences

## Abstract

Groundwater is a vital resource for humans and groundwater dependent ecosystems. Coastal aquifers and submarine groundwater discharge (SGD), both influenced by terrestrial and marine forces, are increasingly affected by climate variations and sea-level rise. Despite this, coastal groundwater resources and discharge are frequently poorly constrained, limiting our understanding of aquifer responses to external forces. We apply traditional and novel time-series approaches using an SGD dataset of previously unpublished resolution and duration, to analyze the dependencies between precipitation, groundwater level, and SGD at a model site (Kīholo Bay, Hawaiʻi). Our objectives include (1) determining the relative contribution of SGD drivers over tidal and seasonal periods, (2) establishing temporal relationships and thresholds of processes influencing SGD, and (3) evaluating the impacts of anomalous events, such as tropical storms, on SGD. This analysis reveals, for example, that precipitation is only a dominant influence during wet periods, and otherwise tides and waves dictate the dynamics of SGD. It also provides time lags between intense storm events and higher SGD rates, as well as thresholds for precipitation, wave height and tides affecting SGD. Overall, we demonstrate an approach for modeling a hydrological system while elucidating coastal aquifer and SGD response in unprecedented detail.

## Introduction

The coupled coastal aquifer-ocean system is a highly dynamic environment at the convergence of land and ocean, where major water and chemical fluxes are driven by the hydrological cycle^1^. Complex biogeochemical interactions modulate inputs to the coastal ecosystem from both land and sea, which are increasingly affected by burdensome levels of anthropogenic influence, such as urban, industrial, and agricultural runoff, in addition to impacts associated with climate change^2,3^. While the importance of preserving coastal water quality has been well documented, many coastal environments and hydrological processes occurring in these systems remain understudied and poorly characterized^4^. Understanding coastal hydrological interactions are key for maintaining sustainable water resources and groundwater dependent ecosystems, particularly in the advent of a changing climate, atmospheric warming, and increasing sea levels.

Coastal aquifers and subsurface flow are insufficiently examined in many locations globally. Groundwater is the largest reservoir of readily available fresh water for drinking water resources, but these aquifers, especially those located along coastlines, are vulnerable to anthropogenic and climatic pressures^5^. For example, groundwater is the primary source of drinking water on Pacific Islands, but changes in rainfall patterns and sea-level rise are leading to decreasing potable water and increasing aquifer salinization^2,5^. Overlapping terrestrial and oceanic processes occurring on differing temporal scales and magnitudes mean it is frequently challenging to analyze interactions of groundwater with other water reservoirs (e.g., precipitation, terrestrial surface runoff, ocean).

Groundwater levels are also influenced by oceanic-atmospheric climate variability such as the El Niño Southeast Oscillation (ENSO) and the Pacific Decadal Oscillation (PDO). The ENSO has three phases (Neutral, El Niño, and La Niña), determined by the Ocean Niño Index (ONI), which is based off sea surface temperature (SST) variability^6^. El Niño (warmer than average SST; ONI > 0.5) and La Niña (cooler than average SST; ONI <  − 0.5) can both have major impacts to local weather patterns and ocean conditions globally^6^. For instance, El Niño is associated with drier conditions in the central Pacific^6^. El Niño is also associated with increased tropical cyclone activity in the Pacific Ocean, which can result in substantial amounts of episodic rainfall^6^. Changes in precipitation, for instance, directly affect groundwater levels as recharge is the major process leading to aquifer replenishment.

Submarine groundwater discharge (SGD), or groundwater that discharges to the coastal ocean, has long been recognized as both a critical resource to coastal ecosystems^7,8^ as well as a vector for pollutants to reach the coastal ocean^9–11^. This is particularly the case for small volcanic islands^12^, where SGD comprises a disproportionate contribution to the water budget relative to land mass. Total SGD is comprised of both fresh and saline SGD. Fresh SGD is primarily driven by terrestrial forces, such as precipitation which influences the vertical hydraulic gradient. In comparison, saline SGD is mostly driven by marine forces such as tidal pumping, wave setup, and seasonal ocean water level fluctuations^4,13^. Critically, SGD can be enriched in chemical constituents that reflect land use, such as nutrients, trace metals, carbon, and pharmaceuticals^14–22^. In a well-balanced, unperturbed system, SGD delivers nutrients and alkalinity that sustain coastal ecosystems^23^. In densely populated areas, however, SGD can be a vector for pollutants leading to poor water quality, coastal eutrophication, and harmful algal blooms^4^.

While SGD is a crucial process in the water cycle, characterization can be challenging given its spatiotemporal heterogeneity and dependence on scale of measurements. For example, field observations of salinity, temperature, and geochemical tracers (e.g., radon, radium) can be used to determine spatial distribution of SGD, whereas hydrological models are typically used to analyze driving forces, temporal variability, and coarser resolution distribution^4,14,24^. Hydrological models can provide insight into water budgets and environmental interactions over longer time scales and provide powerful predictive capacity under future hydrological conditions and dynamics. The successes of these models, however, are typically reliant on an accurate portrayal of hydrogeological parameters^4^.

Understanding changes to SGD and coastal aquifer dynamics under climate change and sea-level rise is increasingly critical. While hydrological models can successfully capture current and predict SGD under future climate scenarios, comprehensive modeling is difficult for complex coastal aquifers with poorly constrained or understood hydrogeology. Furthermore, despite the significance of SGD to coastal water and chemical budgets, few long-term SGD records exist^4^, making it difficult to account for in models. To overcome these challenges, we analyzed observed patterns in SGD during baseline conditions, typical and intense precipitation events, high tides and seasonal ocean levels, and large swell events using long-term high-resolution SGD and hydrological parameter records coupled with big data analyses methods. The approach applied here, while not the goal of this research at present, should also be considered in the broader efforts to upscale SGD estimates both spatially and temporally as it complements hydrological models and puts short-term geochemical point measurements into perspective.

**Figure 1:**
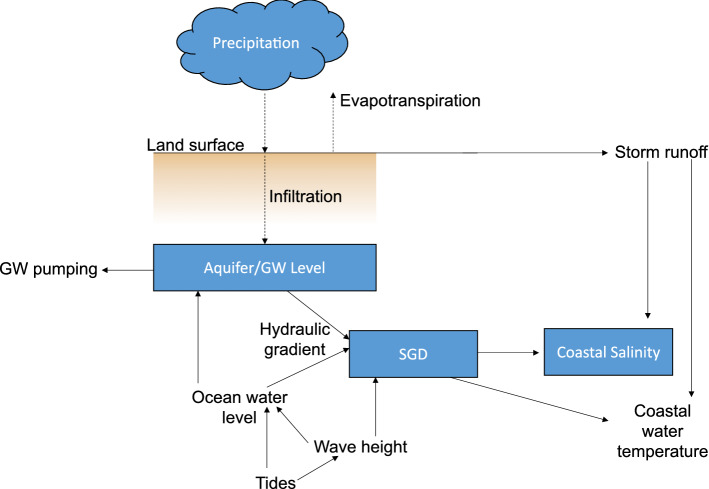
Conceptual flowchart of processes impacting parameters of interest (indicated in blue boxes – coastal salinity, SGD, and groundwater level). The three parameters analyzed were selected because of their direct and environmentally relevant relationships. Ecosystems are greatly dependent on coastal salinity (parameter 1), which at the study site is driven mostly by SGD (parameter 2) and ocean processes. SGD therefore plays an essential ecological role. Groundwater (parameter 3) is the ultimate source of fresh meteoric groundwater in SGD. Additionally, we must acknowledge that marine drivers are also moderating SGD. Tides are not included as a parameter of interest since tides are predictable and instead are used as an explanatory parameter.

Our study system represents a relatively simple directional problem where precipitation and ocean water levels (including tides, swells, seasonal sea level fluctuations) affect the underlying coastal aquifer dynamics (Fig. 1). Here, we employ empirical approaches to understand relationships between atmospheric, terrestrial, and oceanic forces and their role in modifying groundwater levels, SGD, and coastal salinity. We apply this approach to characterize water cycle interactions at a model study site in the Kīholo Aquifer, Hawaiʻi with the objectives of (1) determining the relative contribution of multiple superimposed terrestrial and marine SGD drivers, (2) establishing temporal relationships between processes influencing SGD, such as rainfall, groundwater level, and tides, and (3) evaluate the impact of anomalous events (such as tropical storms) on SGD and its drivers. Our hypotheses include (1) tidally driven forces will have the greatest influence on groundwater levels, SGD, and coastal salinity, (2) precipitation (particularly storm-derived) will precede significant increases in groundwater levels and SGD, and (3) tropical storms and big wave events will lead to increased SGD.

## Study site

**Figure 2:**
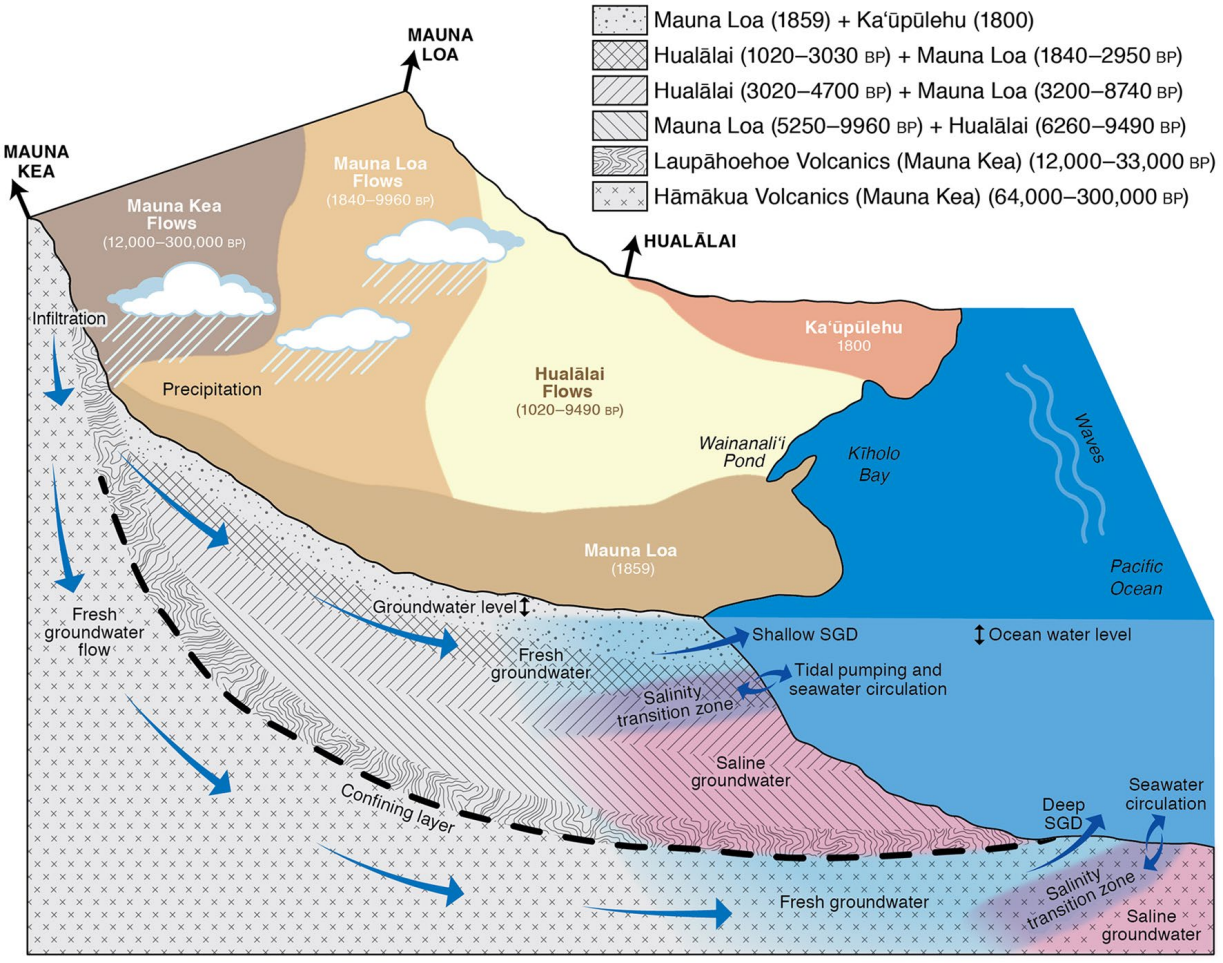
Conceptual cross-section of the study area and processes influencing groundwater levels, SGD, and coastal salinity (not to scale). Locations and ages (years before present, BP) of lava flows are approximated on the surface and with depth according to^26–29^. These historic lava flows are significant as they provide a heterogeneous substrate for groundwater flow that is hard to capture in hydrological models^30^. Precipitation infiltrates into the young, porous basalt and flows in the subsurface before discharging to the coastal ocean as SGD. The assumed location of the subterranean estuary is illustrated by the blue (fresh), purple (brackish), and pink (saline) colors near the discharge zone. These colors are purposefully cut off at a relatively short distance from the shoreline because there is no information on the structure of the aquifer other than the inferred existence of some vertical and horizontal barriers to flow^30^ and deeper conduits^31^. The subsurface geology and hydrogeology in the study area is complex and not well-characterized, but it is hypothesized that there are irregularities and/or a confining layer(s) that impede groundwater flow forcing some SGD to discharge 1–2 km offshore (deep SGD;^31^) in addition to the nearshore, shallow SGD that was the focus of this study^32^. Ocean water level, groundwater level, wave height, tidal pumping, and seawater circulation are all modulated by tidal and seasonal fluctuations. Illustration by Brooks Bays, SOEST Publication Services.

The study was conducted in Wainanaliʻi Pond, located in Kīholo Bay (3.2 km length), on Hawaiʻi Island (19.8583, − 155.9208; Fig. 2; see Supplementary Fig. S1 online). The embayment and pond are flushed by the open ocean and due to freshwater inputs along the shoreline, Wainanaliʻi Pond has estuarine circulation with a sharp stratification and pycnocline at 1 m depth. Tides in the area have a mean diurnal range of 0.65 m^25^. Kīholo Bay is not subject to stream flow and there are no perennial streams in the Kīholo watershed due to the highly permeable lithology.

The Kīholo aquifer is an unconfined basal lens and comprised of interbedded basalt from three different volcanoes, including (from youngest to oldest) Hualālai (elevation = 2,521 m), Mauna Loa (elevation = 4,169 m), and Mauna Kea (elevation = 4,207 m). Kīholo Bay itself is flanked on the surface by two recent lava flows—the 1800 Ka ‘ūpūlehu flow from Hualālai to the south and the 1859 Mauna Loa flow to the north^27,33^ (Fig. 2). Coastal groundwater levels are tidally influenced, even thousands of meters inland from the shore^34^. Within the subsurface numerous structural barriers formed by various types of basaltic lava flows influence, obstruct, and channel groundwater flow, however, these are mostly unspecified^30,35,36^. For example, previous studies have suggested that some Kīholo Aquifer recharge is channeled by a subsurface structural boundary extending from the summit of Hualālai to Puʻu Anahulu^37^ while others have confirmed the presence of deep groundwater conduits^31^, yet none of these features and their hydrogeological roles are well described. Nearshore SGD is well-characterized along this coastline, occurring primarily in the form of point discharge (e.g., discrete springs as opposed to diffuse flow) ranging from 200 to 7000 m^3^/day^38–40^. Mean annual rainfall (based on the 30-year average) to the watershed ranges from 260 to 1200 mm/year^41^, with the most recharge (288,000 m^3^/day) occurring at higher elevations (500 to 2000 m) along the slopes of the volcanoes^42^. Groundwater pumping (33,000 m^3^/day) comprises about 11% of total recharge (Commission on Water Resource Management, *personal communication*).

## Results

### Tidal and seasonal fluctuations

**Figure 3:**
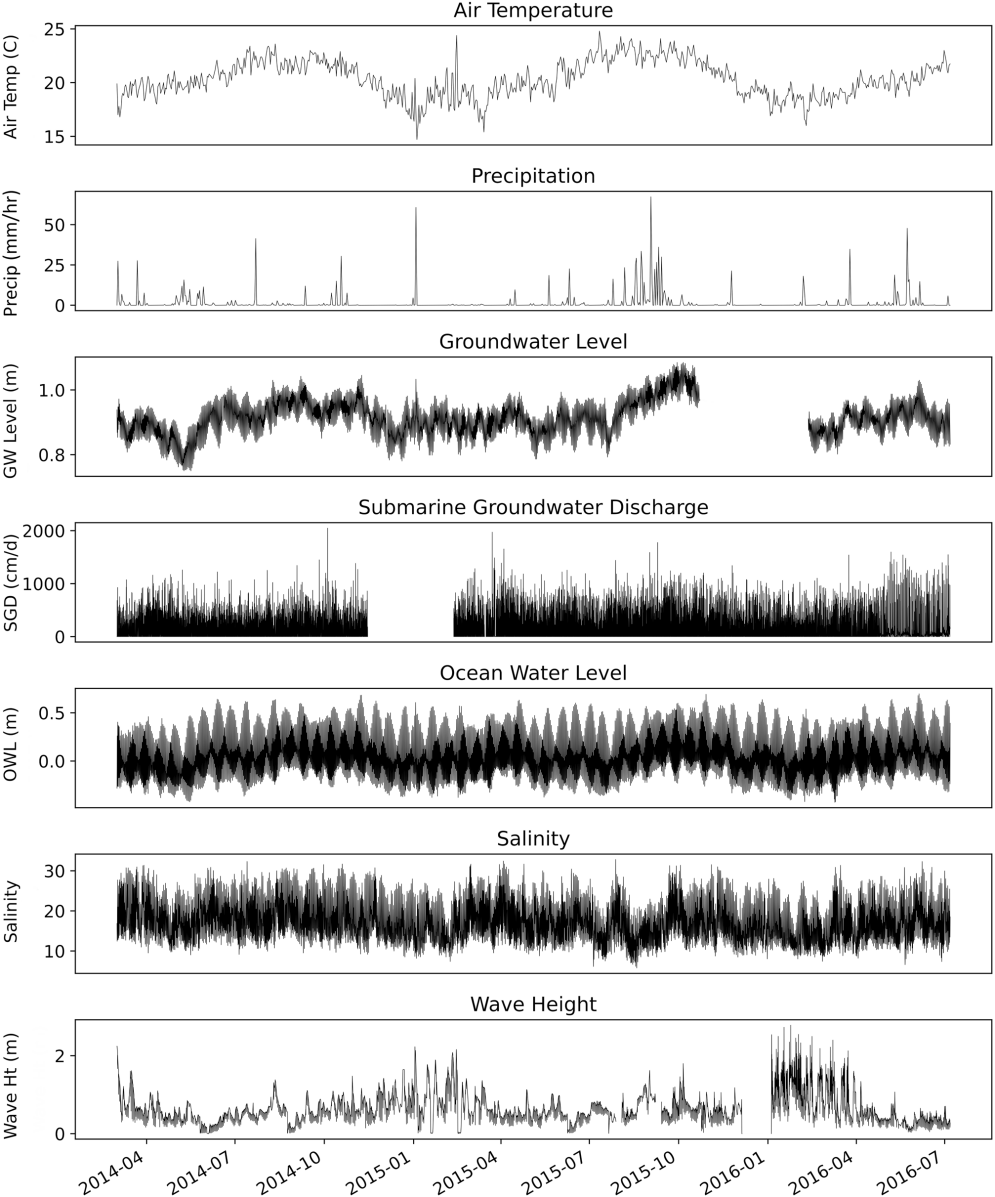
1 h resolution data. Air temperature (^o^C), precipitation (mm/hr), groundwater level (m), SGD (cm/d), ocean water level (m), salinity, and wave height (m) are shown with respect to time. Average uncertainty for SGD was ± 24%.

Seasonality in the time series can be broken down into two main oscillations: tidal and seasonal effects. Parameters that were primarily driven by tides were strongly associated with 6-, 12-, and 25-h oscillations and include ocean water level, groundwater level, salinity, wave height, and SGD (Fig. 3; see Supplementary Figs. S2-S4 online). SGD was greatest at low tide, when the hydraulic gradient (groundwater level minus ocean water level) was at its maximum. Similarly, outside of major precipitation events, salinity was lowest during low tide given the greater influence from SGD. Longer term seasonal effects included air temperature, precipitation, and groundwater level. Wet season (May through October) average air temperature (22 ± 1.2 °C), precipitation (2.1 ± 5.4 mm/hr), and groundwater levels (0.93 ± 0.049 m) were greater than those occurring during the dry season (November through April; 20 ± 0.87 °C, 0.43 ± 1.9 mm/hr, and 0.91 ± 0.029 m, for air temperature, precipitation, and groundwater levels, respectively), although it is worth mentioning that the study period had a significant overlap with an El Niño period.

Groundwater level is both tidally and seasonally modulated as it is influenced by both recharge from precipitation and ocean water level. Because of the porous and highly conductive basalt matrix and groundwater elevations near sea level, groundwater levels are affected by ocean water level^34^. While the groundwater monitoring station was 5.6 km inland from the shore and some signal attenuation is anticipated, ocean water and groundwater levels were positively correlated (R^2^ = 0.54), with a lag of 5 h (see Supplementary Fig. S5online). The lag is distance dependent and expected to decrease closer to the shoreline. However, neither the distance nor the lag affects the 25-h seasonal and longer-term trends analyses.

### Longer term trends

**Figure 4:**
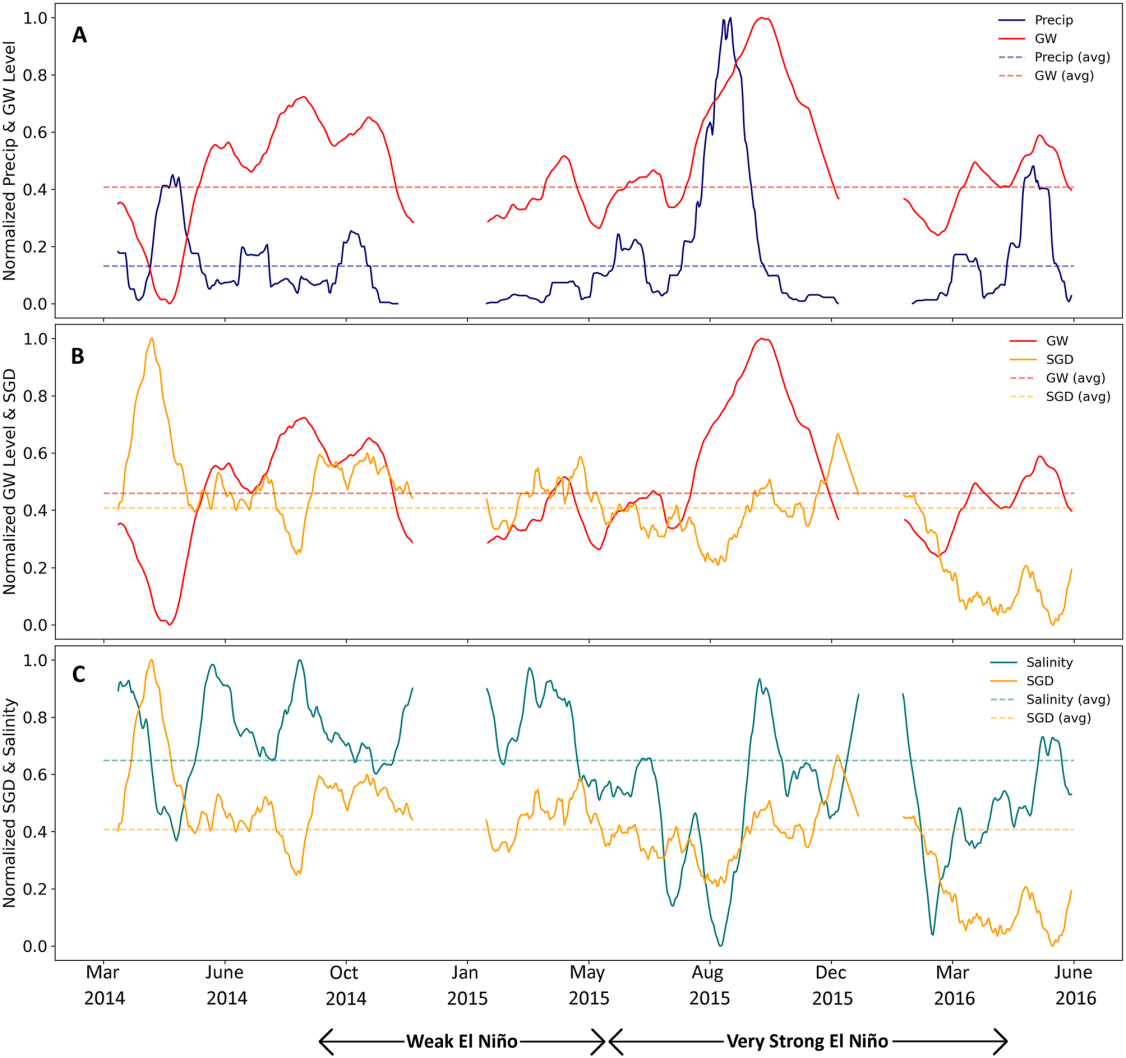
25-h trend component from the time series decomposition. Mean values for each variable are shown in the dashed lines. (**A**) normalized precipitation (blue) and groundwater levels (red), (**B**) normalized groundwater levels (red) and SGD (yellow), and (**C**) normalized SGD (yellow) and coastal salinity (teal) with respect to time. Most of the time series coincided with El Niño (ONI ranging from 0.6 in October through December 2014 to 2.6 in November 2015 through January 2016).

**Figure 5:**
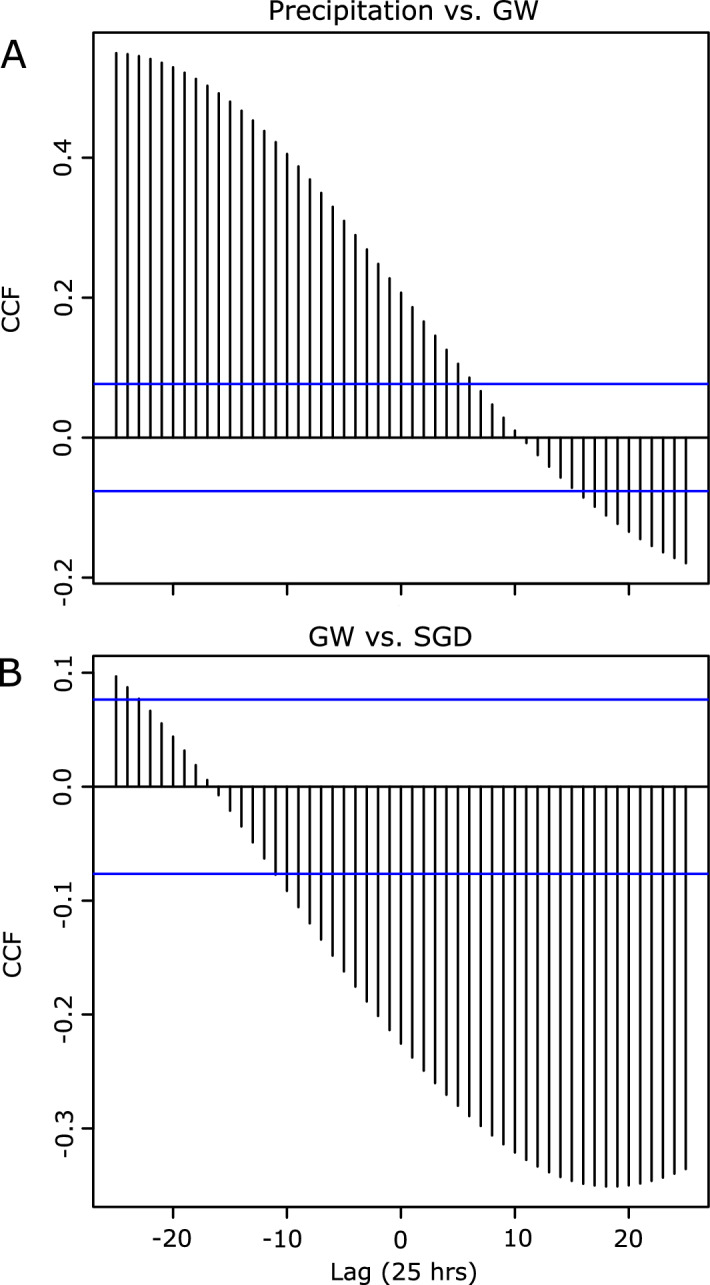
Cross correlation (CCF) between the 25-h resampled trend component for precipitation and groundwater levels (**A**) and groundwater levels and SGD (**B**). The blue lines indicate that the cross correlation is significant at the 95% confidence interval, thus any value outside of the lower and upper bands is significant. The gradual slope on both cross-correlation plots indicates that impacts from precipitation on groundwater (**A**) and groundwater on SGD (**B**) are not instantaneous but accumulate and get pronounced over time.

The longer-term trend component was extracted after removing all tidal and seasonal effects (Fig. 4). Here, we focused on interconnectivity between precipitation, groundwater levels, SGD, and salinity. A cursory examination of these parameters plotted with respect to time shows a clear lagged relationship between precipitation and subsequent groundwater levels, offset by a period of two to four weeks. This relationship was confirmed as significant through cross-correlation, with a maximum R^2^ value at four weeks of 0.55 (Fig. 5). High groundwater levels were also associated with high SGD three weeks later (Fig. 5).

**Figure 6:**
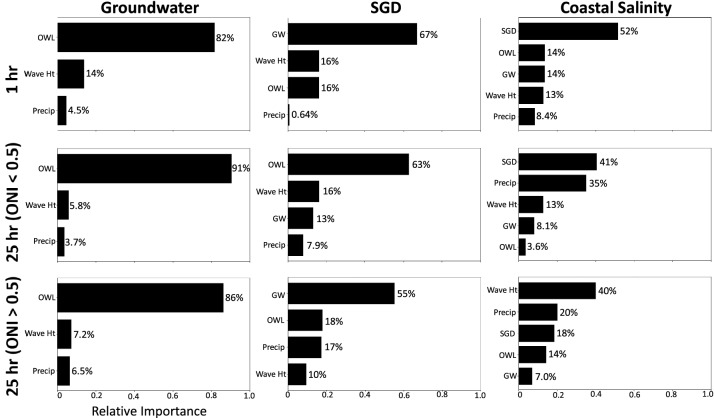
Feature importance calculated using random forest classification conveying relative importance of drivers to groundwater level, SGD, and coastal salinity at 1- and 25-h intervals. 25-h intervals are separated based on periods with El Niño (ONI > 0.5) and without El Niño (ONI < 0.5) conditions present.

Relationships between drivers and parameters of interest were further investigated by quantifying the percentage contribution to each parameter using a random forest classifier to calculate feature importance (Fig. 6). Groundwater level drivers included ocean water level, wave height, and precipitation, and then subsequently SGD included all groundwater drivers in addition to groundwater level, and finally coastal salinity analysis included all SGD drivers, in addition to SGD. At the 1-h temporal resolution, tidally driven effects such as ocean water level, wave height, and SGD were the primary influences on groundwater levels. For SGD, tidally influenced variables were again the greatest contributor at the 25-h temporal resolution when El Niño conditions were not present. During El Niño periods, however, processes such as precipitation had a greater impact on SGD, increasing by 9%. For features influencing salinity at the 25-h resolution, wave height and precipitation had a greater effect during El Niño periods compared to those when El Niño was not ongoing.

## Discussion

Both tidal and seasonal components influence SGD as previously demonstrated in geochemical and hydrological modeling studies^1,3,4,13,14,43^. SGD estimates based off chemical budgets typically represent shorter time scales, whereas hydrological models require adequate knowledge of the aquifer hydrogeology and field observations to create and calibrate the models. Additionally, there are only a few studies that monitor SGD over a sufficient temporal period to discern tidal and seasonal patterns and relationships with its terrestrial and marine driving forces. Here, we propose that SGD observations coupled with precipitation, groundwater level, ocean water level, and wave height data can be used to reveal relationships between SGD driving forces using time series analysis and machine learning methods.

Tidal patterns discerned from the time series decomposition (Fig. 4) and lag analysis (Fig. 5) displayed patterns with 6- (M_4_, shallow water overtides of principal lunar tide), 12-(M_2_ principal lunar semidiurnal tide), and 25-(K_1_, lunar diurnal tide) hour temporal resolutions. Furthermore, the random forest classification (Fig. 6) demonstrated that short-term tidal effects were still the dominant driver for groundwater levels at the resampled 25-h temporal resolution. This makes sense because groundwater levels are influenced by both tidal (ocean water level and wave height), which are present at 24.8 and 28-day periods, and seasonal (precipitation) oscillations, but as precipitation is episodic in nature, the tidal signature will override the seasonal one. Similarly, tidally influenced parameters had the greatest impact to SGD and salinity during periods that did not coincide with extreme events.

Seasonal patterns were associated with fluctuations in precipitation. While precipitation impacts are not instantaneous as demonstrated by the lag analysis (Fig. 5), rainfall, particularly when associated with storm events, led to long-term changes in groundwater levels and SGD (Fig. 4). Over longer time intervals (e.g., comparing 1-h vs. 25-h resolution), precipitation becomes an increasingly important driver to SGD and coastal salinity, as evidenced by the random forest classification (Fig. 6) and studies conducted in other island environments^3^. This corroborates with our lag analyses as precipitation impacts to these components are not instantaneous.

**Figure 7:**
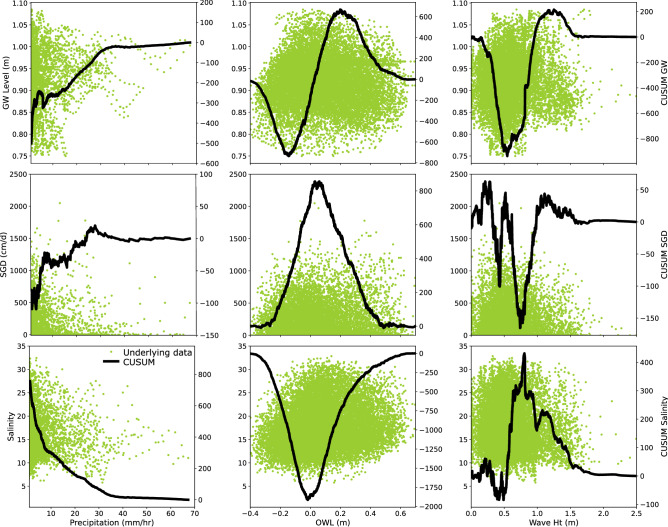
CUSUM driver-response plots (columns = drivers, rows = response). Drivers shown include precipitation rates exceeding 1 mm/hr (mm/hr; column 1), ocean water level (OWL; m with respect to MSL; column 2), and wave height (Wave Ht; m; column 3). These are compared to the following responses: groundwater levels (m; row 1), SGD (cm/d; row 2), and salinity (row 3). The underlying data showing the relationship between x and y are shown in the green dots on the primary y-axis. The driver-response relationship is illustrated with the black line (secondary y-axis), comparing the CUSUM groundwater level, SGD, and salinity vs. precipitation, OWL, and wave height.

Traditional time series approaches, such as decomposition and lag analysis are useful, but may not be able to fully resolve driver-response relationships between environmental parameters given the influence of overlapping processes. These issues were addressed by conducting additional analyses using a CUSUMs approach to examine driver-response relationships between groundwater levels, SGD, coastal salinity, and precipitation, ocean water levels, and wave height (Fig. 7). The CUSUM plot shows the cumulative sum of the underlying (standardized) time series. A positive CUSUM slope arises from the aggregation of above-average underlying values, while a negative CUSUM slope arises from the aggregation of below-average values. Underlying values are more extreme (farther away from the mean) as the CUSUM slope becomes steeper. Overall, periods where precipitation exceeded 1 mm/hr were associated with above average groundwater levels and SGD and below average coastal salinity. While this result is somewhat intuitive, few studies have quantified this effect^1,13^. Further evidence was derived from the resulting CUSUM analysis slope, which can be used as a measure of the amplitude of influence in a driver-response relationship. Precipitation rates between 1 and 4 mm/hr for groundwater (1 to 7 mm/hr for SGD) led to the greatest increase in groundwater and SGD rates (Fig. 7; see Supplementary Table S1 online), where the slope was an order of magnitude greater (72 and 10 × for the relationship between precipitation and groundwater and SGD, respectively) than other precipitation thresholds. The degree of influence declined for precipitation rates between 4 and 34 mm/hr (for groundwater) and 7 and 28 mm/hr (for SGD). Salinity was the lowest at precipitation rates ranging from 1 to 8 mm/hr (slope =  − 65x), and then remained below average for precipitation ranging from 8 to 36 mm/hr (slope = 2.2x). The relatively small sample size for precipitation above 40 mm/hr limits our ability to describe driver-response relationships above this threshold. Overall, slope decreased by 96 to 99% from the first precipitation threshold (range of upper bound: 4–8 mm/hr) to the final precipitation threshold (range of lower bound: 28–36 mm/hr) for groundwater level, SGD, and salinity (see Supplementary Table S1 online). These thresholds indicate the presence of nonlinear response patterns, justifying our multipronged approach.

Tidally driven parameters, such as ocean water level and wave height, had a significant impact on groundwater level, SGD, and salinity. Ocean water levels less than − 0.1 m led to low groundwater levels and between -0.1 to + 0.2 m led to high groundwater levels. Ocean water levels greater than + 0.2 m were typically associated with bimonthly spring tides and had a low groundwater levels, suggesting that there is a threshold for tidal modulation of groundwater, perhaps due to constraints on rock permeability^30^. Ocean water levels less than 0 m were associated with high SGD and low salinity, and the opposite was true for ocean water levels greater than 0 m. This is not surprising as SGD is typically greatest at low tide when the hydraulic gradient is the steepest and one would expect salinity to be the lowest when SGD is greatest. Wave heights less than 0.5 m led to low groundwater levels, between 0.5 and 1.3 m led to high groundwater levels, and then greater than 1.3 m led to low groundwater levels before evening out at 1.5 m (Fig. 7). Wave heights between 1.5 and 2.5 m were typically associated with either tropical storms or big wave events. Similarly, while the relationship between wave height and SGD is less clear-cut when wave height is less than 0.8 m, wave height between 0.8 and 1.2 m led to high SGD, perhaps reflecting additional influence from saline SGD due to increased wave setup, tidal pumping, and recirculating saline water. Salinity had the opposite relationship to wave height compared to SGD, where wave heights between 0.5 and 0.8 m led to high salinity, while those between 0.8 and 1.5 m led to low salinity. Due to the small sample size for wave heights above 1.5 m, we are not able to interpret above this threshold. The impact of wave height on SGD may sound counterintuitive hydrologically because waves can add water into the coastal aquifer during wave set-up^4^, but if the high waves coincide with spring tides, storms, precipitation events, or higher SGD, then the relationship can be explained by the influence from these processes instead.

### Outliers and event detection

Outliers were used for additional analyses and event detection. Univariate outliers (see Supplementary Table S2 online) were examined to look at specific feedback mechanisms but were not used as a basis for outlier detection. Groundwater levels ranged from 0.75 to 1.1 m (average = 0.92 ± 0.053 m with respect to MSL), with outliers occurring for 4 days below the lower bound and 4 days above the upper bound. High groundwater levels occurred during October 2015 and were associated with previous precipitation events sourced from tropical storms in August through September 2015 (see Supplementary Fig. S3 online). Low groundwater levels occurred in May 2014 when ocean water levels were also low. SGD ranged from 0 to 2,000 cm/d (average = 150 ± 240 cm/d), outliers were identified on 3 days below the lower bound and 8 days above the upper bound. Higher SGD rates occurred one to two months after major precipitation events, whereas lower SGD rates were typically associated with lower wave heights, perhaps due to decreased contribution from saline SGD from wave set up. Salinity ranged from 5.8 to 33 during the study period with an average of 18 ± 4.8. Of this, 7 days during the study period were considered outliers below the lower threshold (salinity ranging from 11 to 12) primarily due to high rainfall and 3 were above the upper threshold (salinity ranging from 24 to 25).

Overall, 25 days were identified as multivariate outliers (Table 1). Of these, 8 days were associated with hurricanes, 12 days were associated with big wave events in February through March 2016, and the remaining were associated with rainfall events. The 2015 Central Pacific Hurricane season was the most active on record, with 16 named storms^6^. On August 30, 2015, a record three Category 4 hurricanes (Hurricanes Kilo, Ignacio, and Jimena) were co-occurring east of the international dateline. While this occurrence was anomalous in the historical record, there is evidence that tropical storms will increase in both frequency and intensity around the Hawaiian Islands^44,45^.

**Table 1:** Multivariate outlier events by date, variables above or below average during those dates, and associated cause.

Date	Above average	Below average	Reason
Jul 22, 2014	Precip		Rainfall
Oct 18, 2014	Precip, GW, wave height		Hurricane Ana
Aug 18, 2015	Precip, GW		Hurricane Hilda/Kilo
Aug 23–24, 2015	Precip, air temp, GW, wave ht		Hurricane Kilo
Sep 2–3, 2015	Precip, air temp, GW, wave ht	Salinity	Hurricane Ignacio
Sep 9, 2015	Precip, air temp, GW, wave ht		Hurricane Jimena
Sep 13, 2015	Precip, air temp, GW, wave ht		Hurricane Jimena
Feb 17–24, 2016	Wave ht, OWL		Big wave event
Mar 1–2, 2016	Wave ht	Salinity	Big wave event
Mar 5, 2016	Wave ht	Salinity	Big wave event
Mar 20, 2016	Wave ht		Big wave event
Mar 24, 2016	Precip		Rainfall
May 22–24, 2016	Precip	OWL	Rainfall event coincident with spring tides

Three types of events were identified from the record: heavy rainfall, tropical storms, and big wave events.

Isolating outliers were informative for extracting relationships that may have an even greater influence in the future. For instance, hurricanes were associated with increased precipitation (in addition to higher seasonal air temperatures and wave height), subsequently leading to increased groundwater levels and SGD (Fig. 3). While ocean water levels typically have an inverse relationship with SGD, this effect is amplified during periods of above average wave height (Fig. 4). This is likely because the hydraulic gradient decreases further, leading to decreased SGD. The effects of ENSO and hurricanes on groundwater levels have been documented in other locations globally, such as the mainland United States and India^46,47^. Similar to our results, a model-based study reported SGD, groundwater level, and recharge anomalies that were linked to ENSO for a barrier island in North Carolina, USA^48^.

Climate change in Hawaiʻi is anticipated to lead to decreased monthly average precipitation (but an increase in extreme rainfall events), increasing sea levels, increasing water and air temperature, disruptions to trade wind patterns, and an increase in the hurricane frequency locally^44^. The data collected during this study provides vital information about how precipitation can impact groundwater levels, SGD, and coastal salinity. For instance, an increase in the frequency and intensity of catastrophic rainfall events (and coincident decline in average precipitation) could lead to seasonal changes in the availability of water resources or the magnitude of SGD. All recorded tropical storms that passed near the main Hawaiian Islands during El Niño were associated with outliers in this study, particularly due to changes in precipitation and groundwater levels. Multiple studies have demonstrated that decreased precipitation and increased stress on water resources leads to decreasing fresh SGD, higher sea levels, and salinization of coastal aquifers^49,50^. Major rainfall events also lead to decreasing coastal salinity from storm runoff, which can have negative ecological impacts as biota tend to be sensitive to certain salinities and sediment load.

Both in Hawaiʻi as well as globally, a decrease in fresh SGD and increasing sea levels could have major negative impacts to coastal ecosystem health. Groundwater dependent ecosystems, which rely on lower salinity SGD with optimal nutrient levels, are aggravated by changing precipitation patterns and sea-level rise^51^. Humans have relied on these ecosystems for years—for instance, traditional Hawaiian aquaculture depended on SGD to feed coastal fishponds^52^, such as the one in Kīholo Bay. Beyond looking at this from a human-impact perspective, anthropogenic influence on SGD has also led to coastal eutrophication and harmful algal blooms in for instance, Bermuda^53^, and has been associated with an increase in coral reef disease severity in Guam^54^. These groundwater dependent ecosystems are vulnerable and are posed to face additional challenges in the upcoming decades.

The methods and analyses used here are applicable to other coastal aquifers globally but are particularly useful for areas that lack well-characterized hydrogeology and thus are difficult to describe with a hydrological model. Here, we were able to constrain the temporal relationships between variables, elucidate driver-response relationships, determine which predictors have the greatest influence on a variable, and isolate events to learn more about the relationship between atmospheric, terrestrial, and oceanic processes under conditions that will likely be more common in the future. In this way, our methods provide similar results to a hydrological model without the requirement of a highly monitored subsurface. Given the conditions of the field study, we were unable to separate the fresh and saline SGD fractions due to variations in the radon to salinity ratio and the embayment residence time over longer time periods. While the radon to salinity ratio holds consistent for shorter time periods (weeks to months), seasonal factors such as rainfall or large swell events make it challenging to derive the fresh SGD fraction without very high uncertainties^55^. In another example, urban coastal aquifers frequently experience a “coastal groundwater squeeze” sourced from declining water resources due to increased salinization, overextraction, land subsidence, and pollution^2^. Additional applications could include providing insight to feedback relationships between coastal salinity and precipitation and/or sea-level rise in terms of fish abundance, supply, and diversity. In all, the methodology applied in this study can provide valuable predictive feedback for understanding perturbations to the water cycle in a wide variety of environments.

## Conclusion

This study applies both traditional (e.g., decomposition, cross-correlation) and novel (e.g., random forest classification, CUSUMs analysis) time-series approaches to understand coastal aquifer responses to atmospheric, terrestrial, and oceanic forces at a model study site with poorly characterized hydrogeology. Using these techniques, we were able to establish that there is a two to four week temporal lag between precipitation and groundwater levels. Increases in SGD occurred three weeks following higher groundwater levels. We were also able to demonstrate that tropical storms led to increased SGD due to increased precipitation. The frequency and magnitude of these tropical storms also coincided with a strong El Niño, and the influence of precipitation on SGD increased by over 9% during this period. Yet, outside of anomalous events, tidally driven parameters had the greatest instantaneous influence on groundwater levels, SGD, and coastal salinity.

This research demonstrates the utility of combining field-based hydrological measurements with data-driven approaches, particularly for areas where the underlying aquifer characteristics are difficult to portray in a hydrological model. The methodologies applied in this research can be used to provide further insight into water budgets and management, resources that are increasingly critically threatened under climatic and land-use changes.

## Methods

### Data collection and SGD calculation

Radon data were collected using an autonomous gamma spectrometer called the SGD Sniffer at 0.3 m depth below the surface of the water^32^, which was deployed from March 2014 through July 2016 in Wainanaliʻi Pond. Conductivity, water depth, and water temperature were concurrently logged with a CTD probe (Schlumberger Inc. CTD diver). All data were collected with a 1-h time resolution. Briefly, SGD was calculated using a transient radon mass balance (Eq. (1); see Supplementary Fig. S6 online^56^), where F_SGD_ is the SGD advection rate (m/day), A is the radon activity (Bq/m^3^) measured by the SGD Sniffer, A_gw_, A_ocn_, and A_atm_ refer to radon activities in groundwater, offshore ocean, and the atmosphere, respectively, A_Ra_ is the dissolved ^226^Ra activity, z_pl_ is the plume thickness (m), T refers to the water residence time (days), and F_atm_ and F_mix_ account for radon losses due to atmospheric evasion and lateral mixing (Bq/m^2^/day). Uncertainties were propagated throughout the mass balance calculations.

1 $$F_{SGD} = \frac{{\frac{{\left( {A - A_{ocn } - A_{Ra} } \right) * z_{pl} }}{T} + F_{atm} + F_{mix} }}{{A_{gw} }}$$

F_atm_ was calculated using Eq. (2), where k is the gas transfer coefficient (m/day), a is the radon gas solubility coefficient, and A_atm_ is the radon activity in the atmosphere. Local wind speed (NOAA Tides and Currents, m/s) was used to derive the radon gas transfer and solubility coefficients.

2 $$F_{atm} = k(A - \alpha A_{atm} )$$

Air temperature (^57^, located at 19.7950, − 155.8453, 710 m elevation), precipitation^57^, groundwater level (^58^ USGS site 194,327,156,002,301; located at 19.7210, − 156.0034, 5,570 m from shore), ocean water level (^25^ NOAA Tides & Currents site 1,617,433, with respect to mean sea level, MSL), and wave height^59^ data were obtained from publicly available databases. Additionally, there were several periods where instrumentation was down, and data are missing (See Supplementary Table S3 online). To overcome these periods of missing data, we primarily focused on using time series analysis methods that can accommodate missing data.

The study period partially overlapped with the 2014–2016 El Niño, with an ONI ranging from 0.6 to 2.6^6^. Additionally, the study period coincided with the 2015 Pacific hurricane season, the second most active on record^6^ and the most active within the central Pacific ^6^. In winter of 2016, big wave events also overlapped with the monitoring period.

### Data pre-processing

All analyses were conducted in Python and R. First, data were power transformed by adding 1 and taking the square root so that its distribution approximates the normal distribution more closely. This was done for all analyses except the cumulative sums (CUSUMs) analysis (described below). After this, data were standardized using Eq. (3), where x_i_ refers to the i-th value in the time series, m is the mean, σ is the standard deviation, and Z_i_ represents the standardized value.

3 $$Z_{i} = \frac{{\left( {x_{i} - m} \right)}}{\sigma }$$

Because hydrological processes and interactions may be more apparent on different time scales or are not instantaneous events, we also conducted a second set of analyses using data that were resampled to a 25-h temporal resolution with short-term tidal fluctuations removed. The tidal signal was removed from the 25-h resampled data by calculating a running average over a six-hour interval and subsequently a 25-h interval. The data were then resampled into 25-h time intervals using the median value from each period.

### Time-series analyses

Following initial preprocessing of data, we conducted an additive time series decomposition using moving averages for each variable using *statsmodels*^60^ to derive the trend, seasonal, and residual components. From here, we extracted the de-seasoned trend component for further analyses to look at longer-term relationships. The Augmented Dickey-Fuller Test was used to confirm stationarity of the decomposed components at the 5% level of significance.

Outliers were detected using two different methods—univariate outliers were identified using the 1.5 interquartile range (IQR) rule and multivariate outliers were detected by calculating the Mahalanobis Distance. The univariate outliers were not removed from the data, but instead were used to observe individual predictor variance and trends. The multivariate outliers were removed from the data and set aside for additional event classification and analysis. In most cases, however, there was substantial overlap between outlier date and time identified using either method. To detect univariate outliers, the 1.5 IQR rule was used, where a data point is considered an outlier if it is less than Q1 – 1.5 * IQR or greater than Q3 + 1.5 * IQR, where Q1 and Q3 refer to the first and third quantile, respectively.

Multivariate outliers were detected by calculating the Mahalanobis Distance with the de-seasoned dataset using Eq. (4) (MD;^61^, where the multivariate vector is represented by $\overline{x}$, with mean $\overline{\mu }$, and S is the covariance matrix. For each time point, p-values were calculated for the resulting MD by calculating the *χ*^2^ value for each MD. Outliers were identified when the resulting p-value was less than 0.001.

4 $$MD = \sqrt {\left( {\overline{x} - \overline{\mu }} \right)^{T} S^{ - 1} \left( {\overline{x} - \overline{\mu }} \right)}$$

Temporal lags between precipitation, groundwater, and SGD were assessed for the 25-h resampled data using the cross-correlation function (CCF) in R. Cross-correlation plots can indicate either lagged or instantaneous relationships between two variables. The estimated lag time was determined as the lag with the greatest R^2^.

To establish the relative influence of each driver, we conducted a random forest classification to extract feature importance for groundwater levels, SGD, and coastal salinity. This analysis was conducted using the *scikit-learn RandomForestRegressor*^62^ in Python for both 1-h resolution and 25-h resampled data. Feature importance was examined during El Niño conditions by constructing separate random forest classifiers for data occurring when the ONI was greater than 0.5 (indicating El Niño conditions) and less than 0.5 (indicating El Niño conditions were not present).

CUSUMs analysis was used to evaluate driver-response relationships. This method can help resolve variable relationships in complex environmental time series data with missing intervals^63^. To quantify driver-response relationships, data were first standardized using Eq. (2). After data were standardized, driver (precipitation, ocean water level, and wave height) and response (groundwater levels, SGD, and salinity) relationships were quantified. For each driver-response relationship (e.g., precipitation impact on groundwater levels), driver data were first organized in ascending order. Then the CUSUM for the *i*th observation for the reordered response variable, s_i_, was calculated using Eq. (5).

5 $$s_{i} = z_{i} + s_{i - 1}$$

The standardized data, z_i_, have zero mean and unit variance (m = 0, σ = 1). When the z_i_ value is positive, the underlying value, x_i_, is greater than the mean. Similarly, when z_i_ is negative, then x_i_ is less than the mean. The slope of the CUSUM trend is an indication of the sign and magnitude of the x_i_ values: a steep positive slope is the result of large positive deviations of x_i_ from its mean, and a steep negative slope is the result of large negative deviations of x_i_ from its mean. CUSUM analysis can be used to examine relationships between variables^63^.

## Supplementary Information

Supplementary Information.

## Data Availability

Data are available from the Hydroshare Repository at 10.4211/hs.7062dca5d72f42e193c0cee9bc1cc2bc.

## References

[1] Santos IR (2009). Extended time series measurements of submarine groundwater discharge Tracers (222Rn and CH4) at a coastal site in Florida. Mar. Chem..

[2] Michael HA, Post VEA, Wilson AM, Werner AD (2017). Science, society, and the coastal groundwater squeeze. Water Resour. Res..

[3] Adyasari D, Montiel D, Mortazavi B, Dimova N (2021). Storm-driven fresh submarine groundwater discharge and nutrient fluxes from a barrier island. Front. Mar. Sci..

[4] Taniguchi M (2019). Submarine groundwater discharge: updates on its measurement techniques, geophysical drivers, magnitudes, and effects. Front. Environ. Sci..

[5] IPCC. Global warming of 1.5°C. An IPCC Special Report on the impacts of global warming of 1.5°C above pre-industrial levels and related global greenhouse gas emission pathways, in the context of strengthening the global response to the threat of climate change. *Ipcc - Sr15* **2**, 17–20 (2018).

[6] National Weather Service (NWS). Climate Prediction Center. https://www.cpc.ncep.noaa.gov/products/expert_assessment/ (2021).

[7] Duarte TK, Pongkijvorasin S, Roumasset J, Amato D, Burnett K (2010). Optimal management of a Hawaiian coastal aquifer with nearshore marine ecological interactions. Water Resour. Res..

[8] Boulton AJ (2020). Editorial: Conservation of groundwaters and their dependent ecosystems: Integrating molecular taxonomy, systematic reserve planning and cultural values. Aquat. Conserv. Mar. Freshw. Ecosyst..

[9] Hwang DW, Lee YW, Kim G (2005). Large submarine groundwater discharge and benthic eutrophication in Bangdu Bay on volcanic Jeju Island, Korea. Limnol. Oceanogr..

[10] Lee YW, Hwang DW, Kim G, Lee WC, Oh HT (2009). Nutrient inputs from submarine groundwater discharge (SGD) in Masan Bay, an embayment surrounded by heavily industrialized cities, Korea. Sci. Total Environ..

[11] Amato DW, Smith CM, Duarte TK (2018). Submarine groundwater discharge differentially modifies photosynthesis, growth, and morphology for two contrasting species of Gracilaria (Rhodophyta). Hydrology.

[12] Zektser, I.S. & Everett, L.G. Groundwater and the Environment: Applications for the Global Community. (Lewis Publishers, 2000).

[13] Santos IR, Burnett WC, Chanton J, Dimova N, Peterson RN (2009). Land or ocean?: Assessing the driving forces of submarine groundwater discharge at a coastal site in the gulf of mexico. J. Geophys. Res. Ocean..

[14] Moore WS (2010). The Effect of Submarine Groundwater Discharge on the Ocean. Ann. Rev. Mar. Sci..

[15] Valiela I (1990). Transport of groundwater-borne nutrients from watersheds and their effects on coastal waters. Biogeochemistry.

[16] Rodellas V (2015). Submarine groundwater discharge as a major source of nutrients to the Mediterranean Sea. Proc. Natl. Acad. Sci. U. S. A..

[17] Jeong J, Kim G, Han S (2012). Influence of trace element fluxes from submarine groundwater discharge (SGD) on their inventories in coastal waters off volcanic island, Jeju, Korea. Appl. Geochemistry.

[18] Kim I, Kim G (2015). Role of colloids in the discharge of trace elements and rare earth elements from coastal groundwater to the ocean. Mar. Chem..

[19] Webb JR (2019). Groundwater as a source of dissolved organic matter to coastal waters: Insights from radon and CDOM observations in 12 shallow coastal systems. Limnol. Oceanogr..

[20] Szymczycha B, Borecka M, Białk-Bielińska A, Siedlewicz G, Pazdro K (2020). Submarine groundwater discharge as a source of pharmaceutical and caffeine residues in coastal ecosystem: Bay of Puck, southern Baltic Sea case study. Sci. Total Environ..

[21] McKenzie T (2020). Submarine groundwater discharge: A previously undocumented source of contaminants of emerging concern to the coastal ocean (Sydney, Australia). Mar. Pollut. Bull..

[22] McKenzie T, Habel S, Dulai H (2021). Sea-level rise drives wastewater leakage to coastal waters and storm drains. Limnol. Oceanogr. Lett..

[23] Fujita K (2019). Increase in fish production through bottom-up trophic linkage in coastal waters induced by nutrients supplied via submarine groundwater. Front. Environ. Sci..

[24] Sawyer AH, David CH, Famiglietti JS (2016). Continental patterns of submarine groundwater discharge reveal coastal vulnerabilities. Science (80-.).

[25] National Oceanic and Atmospheric Administration (NOAA). Tide Station 1617433 Kawaihae, HI. https://tidesandcurrents.noaa.gov/stationhome.html?id=1617433 (2021).

[26] Rowland SK, Walker GPL (1990). Pahoehoe and aa in Hawaii: volumetric flow rate controls the lava structure. Bull. Volcanol..

[27] Kauahikaua J, Cashman K, Clague D, Champion D, Hagstrum J (2002). Emplacement of the most recent lava flows on Hualālai Volcano Hawai’i. Bull. Volcanol..

[28] Hammer JE, Coombs ML, Shamberger PJ, Kimura JI (2006). Submarine sliver in North Kona: A window into the early magmatic and growth history of Hualalai Volcano, Hawaii. J. Volcanol. Geotherm. Res..

[29] Sherrod, B. D. R., Sinton, J. M., Watkins, S. E., Brunt, K. M. & Survey, U. S. G. Geologic Map of the State of Hawai‘i, Sheet 4 - Island of Moloka`i. *USGS Open File Rep.* 2007 (2007).

[30] Oki, D. S. Geohydrology and numerical simulation of the ground-water flow system of Kona, island of Hawaii. *U.S. Geol. Surv. Water-Resources Investig. Rep. 99–4073* 70 p (1999).

[31] Attias E, Thomas D, Sherman D, Ismail K, Constable S (2020). Marine electrical imaging reveals novel freshwater transport mechanism in Hawai’i. Sci. Adv..

[32] Dulai H (2016). Autonomous long-term gamma-spectrometric monitoring of submarine groundwater discharge trends in Hawaii. J. Radioanal. Nucl. Chem..

[33] Wolfe E, Morris J (1990). New geologic map of the Island of Hawaii. Trans. Geotherm. Resour. Counc..

[34] Rotzoll K, Fletcher CH (2013). Assessment of groundwater inundation as a consequence of sea-level rise. Nat. Clim. Chang..

[35] Bauer, G. R. A study of the ground-water conditions in North and South Kona and South Kohala Districts, Island of Hawaii, 1991–2002. 95 (2003).

[36] Tillman FD, Oki DS, Johnson AG, Barber LB, Beisner KR (2014). Investigation of geochemical indicators to evaluate the connection between inland and coastal groundwater systems near Kaloko-Honokōhau National Historical Park, Hawai’i. Appl. Geochem..

[37] Tillery, S., El-Kadi, A., Conceptual model for Kiholo Bay watershed and Kaloko-Honokohau National Historical Park, Hawaii. *EPSCoR Hawaiʻi Report* 100 p (2012).

[38] Waters, C. A. Variability in Submarine Groundwater Discharge Composition and the Fate of Groundwater Delivered Nutrients at Kīholo Bay and Honokōhau Harbor, North Kona District, Hawaiʻi. (University of Hawaiʻi at Mānoa, 2015).

[39] Dimova NT, Swarzenski PW, Dulaiova H, Glenn CR (2012). Utilizing multichannel electrical resistivity methods to examine the dynamics of the fresh water-seawater interface in two Hawaiian groundwater systems. J. Geophys. Res. Ocean..

[40] Peterson RN (2008). Radon and radium isotope assessment of submarine groundwater discharge in the Yellow River delta, China. J. Geophys. Res. Ocean..

[41] Giambelluca TW (2013). Online rainfall atlas of Hawai’i. Bull. Am. Meteorol. Soc..

[42] Engott, J.A. A water-budget model and assessment of groundwater recharge for the Island of Hawai'i: *U.S. Geol. Surv. Scientific Investig. Rep. 2011–5078*, p 53 (2011).

[43] Taniguchi M, Ono M, Takahashi M (2014). Multi-scale evaluations of submarine groundwater discharge. IAHS-AISH Proc. Rep..

[44] Murakami H, Wang B, Li T, Kitoh A (2013). Projected increase in tropical cyclones near Hawaii. Nat. Clim. Chang..

[45] Chu JE (2020). Reduced tropical cyclone densities and ocean effects due to anthropogenic greenhouse warming. Sci. Adv..

[46] Gurdak JJ (2007). Climate variability controls on unsaturated water and chemical movement, High Plains Aquifer, USA. Vadose Zone J..

[47] Rishma C, Katpatal YB (2018). ENSO modulated groundwater variations in a river basin of Central India. Hydr. Res..

[48] Anderson WP, Emanuel RE (2010). Effect of interannual climate oscillations on rates of submarine groundwater discharge. Water Resour. Res..

[49] Ericson JP, Vörösmarty CJ, Dingman SL, Ward LG, Meybeck M (2006). Effective sea-level rise and deltas: Causes of change and human dimension implications. Glob. Planet. Change.

[50] Ferguson G, Gleeson T (2012). Vulnerability of coastal aquifers to groundwater use and climate change. Nat. Clim. Chang..

[51] Moosdorf N, Oehler T (2017). Societal use of fresh submarine groundwater discharge: An overlooked water resource. Earth-Science Rev..

[52] Costa-Pierce BA (1987). Aquaculture in Ancient Hawaii. Bioscience.

[53] Lapointe BE, O’Connell J (1989). Nutrient-enhanced growth of Cladophora prolifera in harrington sound, bermuda: Eutrophication of a confined, phosphorus-limited marine ecosystem. Estuar. Coast. Shelf Sci..

[54] Redding JE (2013). Link between sewage-derived nitrogen pollution and coral disease severity in Guam. Mar. Pollut. Bull..

[55] Hudson, C. & Dulai, H. Variability of Submarine Groundwater Discharge During Spring and Neap Tidal Cycles on the Kona Coast, Hawaii. *Geological Society of America Cordilleran Section Meeting*. Honolulu, Hawaiʻi. 23-25 May 2017.

[56] Dulaiova H, Camilli R, Henderson PB, Charette MA (2010). Coupled radon, methane and nitrate sensors for large-scale assessment of groundwater discharge and non-point source pollution to coastal waters. J. Environ. Radioact..

[57] RAWS USA Climate Archive. Puu Waawaa Hawaii. https://raws.dri.edu/cgi-bin/rawMAIN.pl?hiHPUW (2021).

[58] United States Geological Survey (USGS). USGS 194327156002301 8–4360–01 Kalaoa N. Kona (W12–11), HI. https://waterdata.usgs.gov/nwis/uv?site_no=194327156002301 (2021).

[59] Cheung, K.F. WaveWatch III (WW3) Hawaii Regional Wave Model. http://pacioos.org/metadata/ww3_hawaii.html (2011).

[60] Seabold, S. & Perktold, J. Statsmodels: Econometric and Statistical Modeling with Python. *Proc. 9th Python Sci. Conf.* (2010). 10.25080/majora-92bf1922-011

[61] Mahalanobis P (1936). On the generalised distance in statistics. Proc. Natl. Inst. Sci. India.

[62] Pedregosa F (2011). Scikit-learn: Machine Learning in Python. J. Mach. Learn. Res..

[63] Regier P, Briceño H, Boyer JN (2019). Analyzing and comparing complex environmental time series using a cumulative sums approach. MethodsX.

