# Supplementary material for: Traditional and novel time-series approaches reveal submarine groundwater discharge dynamics under baseline and extreme event conditions
Source: Sci Rep. 2021 Nov 19;11:22570. doi: 10.1038/s41598-021-01920-0 (PMC8604958; doi:10.1038/s41598-021-01920-0)
Supplement: Supplementary file 1 — Supplementary Information. [file 41598_2021_1920_MOESM1_ESM.pdf]

Supplementary Material for "Traditional and Novel Time-Series Approaches Reveal Submarine Groundwater Discharge Dynamics Under Baseline and Extreme Event Conditions"

Authors: Tristan McKenzie (tristan.mckenzie@gu.se), Henrietta Dulai, Peter Fuleky

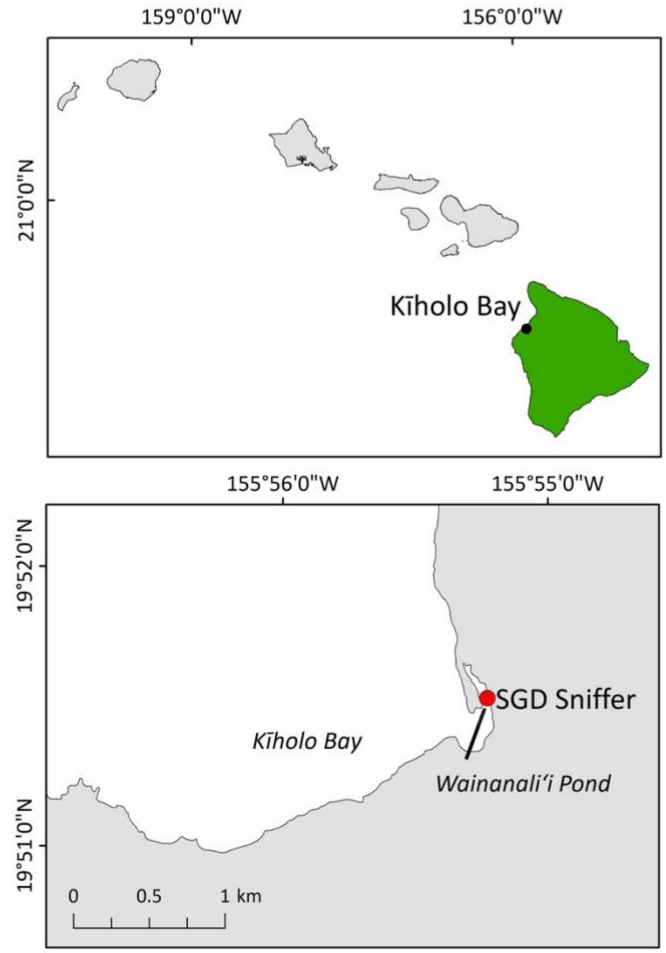

Figure S1. Study location map. The study took place in Wainanali‘i Pond in Kīholo Bay, on Hawai‘i Island. The location of the SGD Sniffer is indicated by the red dot.

Table S1. Summary of linear fit for CUSUM relationships between precipitation and groundwater (GW), SGD, and salinity. Precipitation thresholds (mm/hr) are based off changes in slope. The linear fit results are described by the slope and slope angle. Goodness of fit is described by the R^2 value. The slope % change describes the percentage change between the previous precipitation threshold and the threshold listed in the same row.

Relationship	Precip (mm/hr)	Slope (angle)	Slope R^2	Slope % change
Precipitation & GW	1-4	71.59x (9°)	0.73	
	4 – 34	8.210x (61°)	0.82	89%
	>34	0.5953x (87°)	0.82	93%
Precipitation & SGD	1-7	10.47x (17°)	0.73	
	7 – 28	2.176x (57°)	0.67	79%
	>28	-0.3763x (97°)	0.48	85%
Precipitation & Salinity	1-8	-64.82x (165°)	0.94	
	8 – 36	-11.67x (127°)	0.99	82%
	>36	-0.6326x (92°)	0.92	95%

Table S2. Summary statistics of hourly time series data for the variables of interest - including SGD (cm/d), precipitation (precip; mm/hr), groundwater (GW; m), ocean water level (OWL; m), wave ht (m), and coastal salinity used in this study. Values for the minimum, maximum, mean ± standard deviation, median ± IQR, and lower and upper thresholds based on the 1.5 IQR rule are given for each variable. univariate # of days below or above threshold/bound. Although SGD is expressed in cm/day, the maximum represents the instantaneous (1-hour) flux.

	Min	Max	Mean	Median	Daily Lower Bound	Daily Upper Bound	# Days Below Lower Bound	# Days Above Upper Bound
SGD (cm/d)	0	2100	150 ± 230	20 ± 220	46.83	251.98	3	8
Precip (mm/hr)	0	67	1.6 ± 4.8	0 ± 0.62	0	2.017	0	67
GW (m)	0.75	1.1	0.92 ± 0.053	0.92 ± 0.73	0.81	1.038	4	4
OWL (m)	-0.43	0.70	0.074 ± 0.21	0.052 ± 0.23	-0.11	0.262	2	0
Wave Ht (m)	0.0010	2.5	0.578 ± 0.290	0.530 ± 0.32	0	1.16	0	19
Salinity	5.8	33	17.6 ± 4.82	16.9 ± 6.85	12	24	7	3

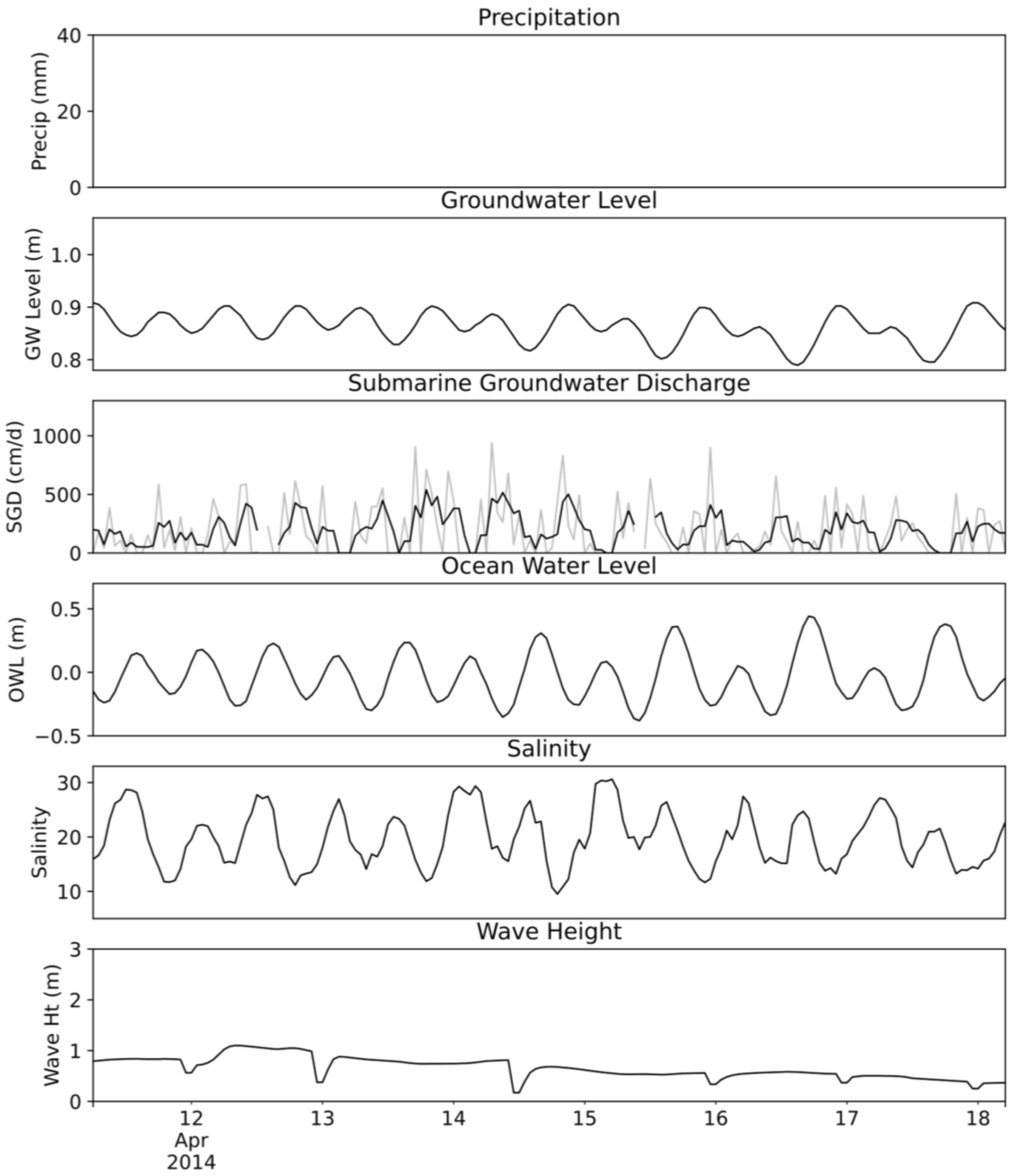

Figure S2. Zoomed in 1-hr resolution time-series exemplifying a period without rainfall (April 11-18, 2014). During this period, mean groundwater levels, SGD, and salinity were 0.86 ± 0.029 m, 190 ± 230 m^3/d, and 19.7 ± 5.17, respectively. For SGD, the underlying hourly data are shown in gray with the 3-hr moving average in black. The seesaw pattern in SGD data is an artifact of the radon model as it relies on changes in radon inventory affected by mixing (small scale eddies and currents) and thus the 3-hr moving average more closely represents actual discharge patterns.

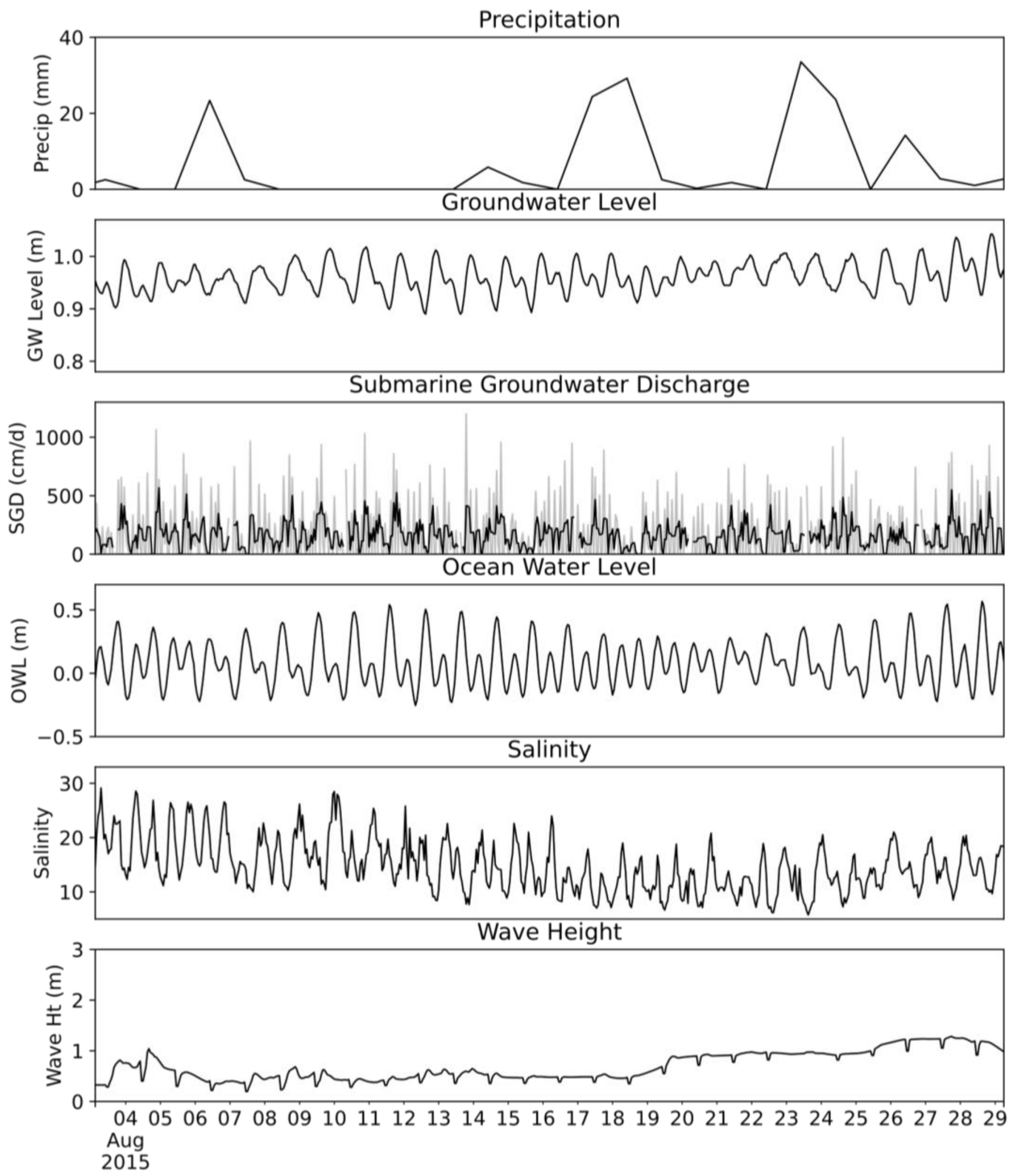

Figure S3. Zoomed in 1-hr resolution time-series exemplifying a period with high rainfall (mean = 6.5 ± 9.0 mm/hr) associated with nearby hurricanes in the Pacific Ocean (August 3-19, 2015). During this period, mean groundwater levels, SGD, and salinity were 0.96 ± 0.029 m, 160 ± 250 m^3/d, and 14.7 ± 4.79, respectively. The high rainfall led to increased groundwater levels and decreased salinity. For SGD, the underlying 1-hr resolution data are shown in gray with the 3-hr moving average in black to remove artifacts from the radon model and more closely represent groundwater discharge patterns.

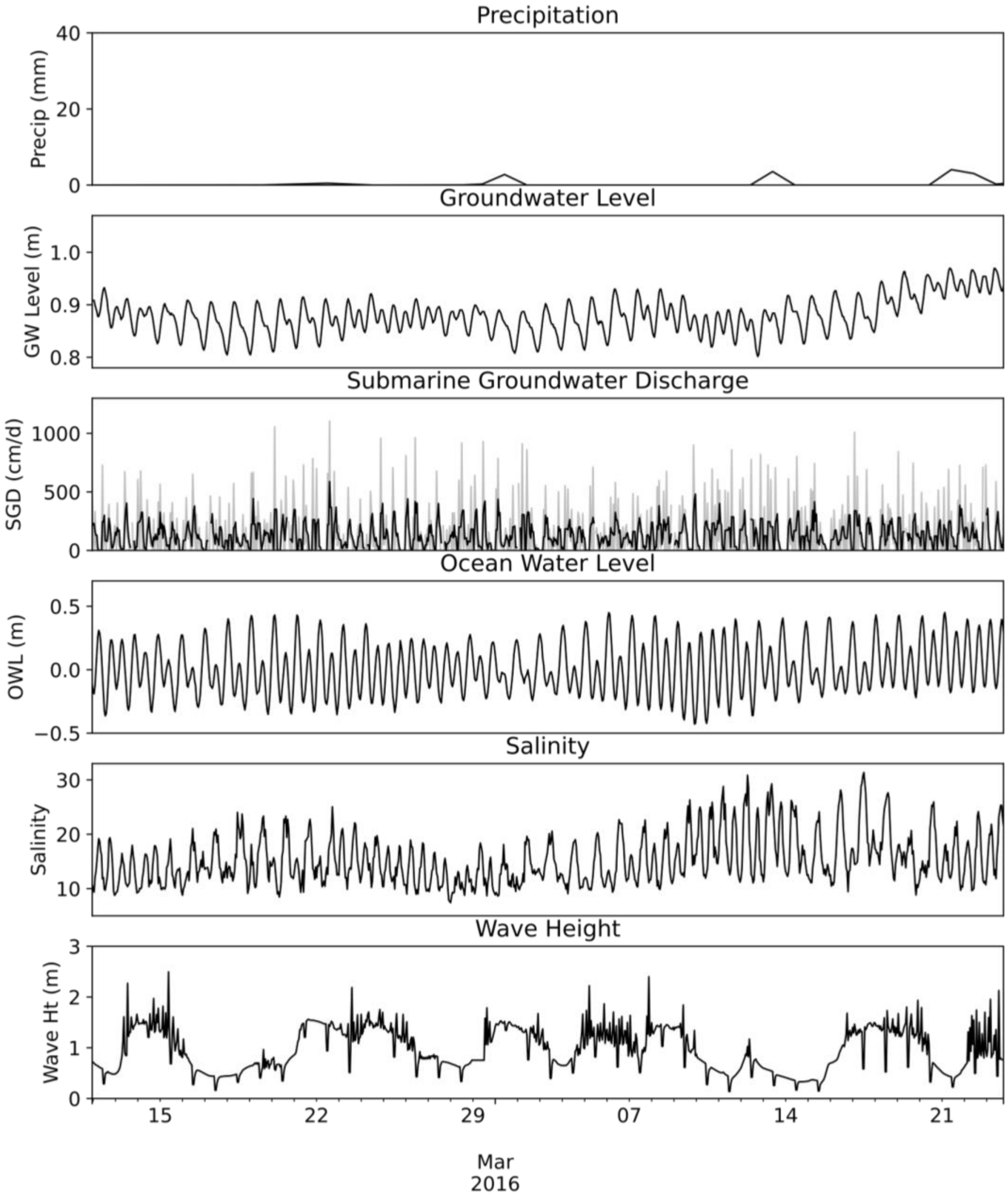

Figure S4. Zoomed in 1-hr resolution time-series exemplifying a period with high wave height (mean = 0.96 ± 0.43 m) associated with big wave events (February 12 - March 25, 2015). During this period, mean groundwater levels, SGD, and salinity were 0.88 ± 0.034 m, 130 ± 210 m^3/d, and 15.3 ± 4.57, respectively. SGD data are shown with 1-hr resolution (gray) and the 3-hour moving average (black) to remove artifacts of the radon model.

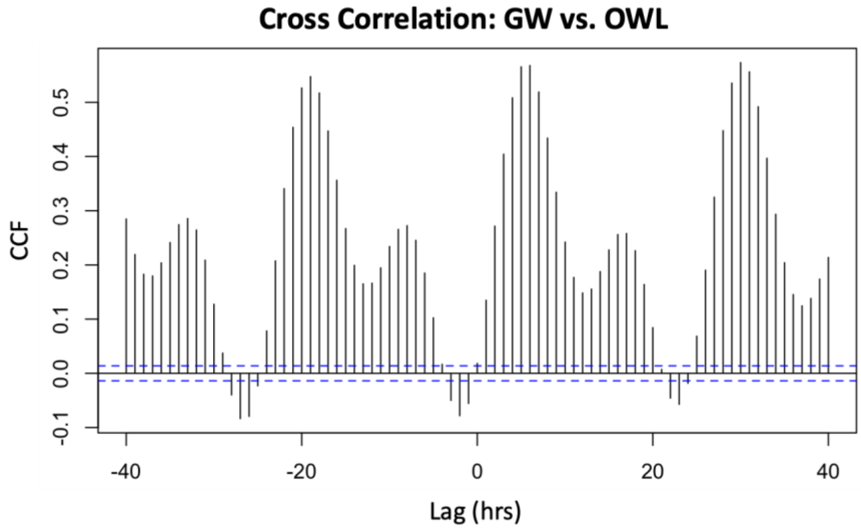

Figure S5. Cross-correlation (CCF) between hourly groundwater (GW) and ocean water levels (OWL). The 95% confidence interval is indicated by the blue dashed lines. Groundwater and ocean water levels are most strongly correlated ($R^2 = 0.54$) at t = +5 hours.

Table S3. Time periods with missing data. Variables with missing data include SGD, groundwater levels, and wave height.

Time Period	Variable with missing data
Nov. 23, 2014 – Feb. 18, 2015	SGD
Oct. 22, 2015 – Feb. 11, 2016	Groundwater
Dec. 5, 2015 – Jan. 4, 2016	Wave height

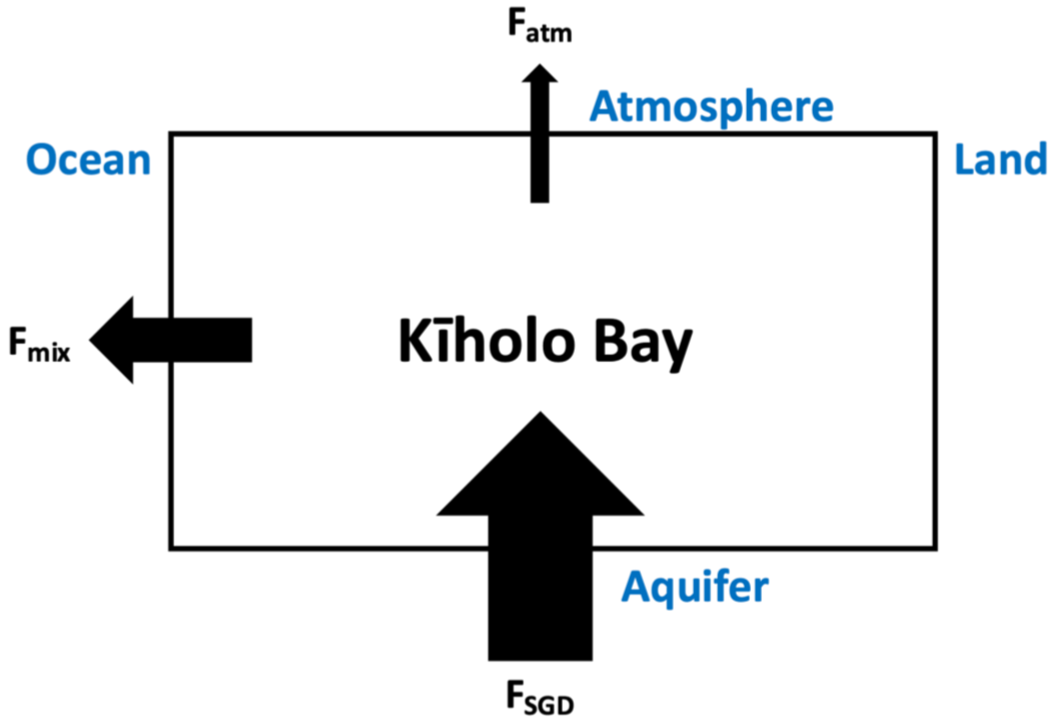

Figure S6. Schematic of the radon mass balance model used to derive SGD fluxes. SGD is calculated as the radon flux from F_{SGD} minus radon losses associated with ocean mixing (F_{mix}) and atmospheric evasion (F_{atm}). Arrows indicate the relative magnitude of each flux. Details on the instrument, measurement geometry, and the applied methods to derive SGD can be found in [40].
